# A portable survivorship care plan: a tool that helps educate and improve knowledge in childhood cancer survivors

**DOI:** 10.1007/s00520-020-05422-z

**Published:** 2020-04-23

**Authors:** Patricia Murphy, Alyssa Levine, Tanya Lerma, Sabrina Young, Jimmy Hwang, Robert Goldsby

**Affiliations:** Pediatric Oncology, UCSF Benioff Children’s Hospital, 550 16th Street, 4th Floor, San Francisco/Oakland, CA 94158 USA

**Keywords:** Childhood cancer survivors, Long term follow-up, Patient education, Survivorship

## Abstract

**Purpose:**

There is a growing population of survivors of childhood cancer at risk for late effects that can affect their overall quality of life. There is evidence that they have inadequate knowledge about their diagnosis, treatment, and subsequent late effects. A randomized study was conducted to determine if a portable credit card–sized plastic card, the “Survivor Healthcare Passport,” improved the survivor’s knowledge of diagnosis, treatment, risks, and follow-up care. The study included 126 patients 2 years post-end of cancer treatment and took place at the UCSF Benioff Children’s Hospital Survivorship Clinic.

**Methods:**

Patients attending the UCSF Survivorship clinic were randomized to receive or not receive a passport at their first survivorship clinic visit. Each groups’ knowledge of diagnosis, treatment history, and follow-up needs was assessed at three time points with a questionnaire.

**Results:**

Patients who received the passport distributed immediately after their visit demonstrated improved and sustained knowledge compared with survivors who did not receive the passport until more than 4 months later.

**Conclusion:**

Enhancing a survivor’s knowledge is an important endeavor and a continual challenge for practitioners in survivorship clinics. This portable educational tool helps improve patient knowledge of their cancer, therapy, and follow-up needs. By providing a tangible card that is quick and easy to access, survivors have access to their treatment late effects and follow-up needs that can also be shared with other healthcare providers.

## Introduction

There is increasingly more attention on the well-being of the growing population of childhood cancer survivors and treating chronic conditions related to their prior cancer therapy [1]. The number of childhood cancer survivors is increasing, with more than 84% of children diagnosed with cancer expected to survive at least 5 years [2]. In the USA, approximately 1 in 530 adults ages 20–39 is a survivor of childhood cancer [2]. Survivors have been shown to be at increased risk for late effects affecting their function and quality of life such as cardiovascular, pulmonary, endocrine, fertility, and musculoskeletal disorders to name a few [3]. Survivors have twice the disease-burden at age 45 years when compared with the general population, with an excess risk of 7 or more chronic health conditions, 2 of which being severe to life-threatening [3]. With increasing survival rates, managing these late effects is a significant concern.

Enhancing a survivor’s knowledge is an important and necessary endeavor for a successful survivorship clinic. As many survivors of childhood cancer are treated at a young age, many will not remember specifics of their diagnosis and treatment [4]. However, lack of knowledge may be independent of age at diagnosis and educational level [5]. One study found that only 50% of childhood cancer survivors were able to list one or more specific drugs they received [6]. For older children, potential psychological trauma may influence the ability to recall treatment details [7]. Additionally, the therapy itself may impact a survivor’s cognitive ability to retain their therapy details and follow-up needs. It is well known that patients who have CNS tumors receiving cranial radiation treatment and/or receiving neurotoxic chemotherapy are at risk for neurocognitive impairments [8]. There is also evidence that long-term childhood cancer survivors who did not receive neurotoxic treatment can have neurocognitive deficits related to other health problem sequelae [9]. Other studies have shown that patients with acute lymphoblastic leukemia treated without radiation may also have neurocognitive consequences [10, 11].

For childhood cancer survivors, there is evidence that they have inadequate knowledge about their diagnosis, treatment, and subsequent late effects [12]. In a study on unmet needs by adult survivors of childhood cancer, survivors reported that many aspects of follow-up care were unmet, including emotional and coping, along with a lack of cancer and treatment information, health care, and surveillance follow-up needs [13]. It has been found that the majority of survivors and parents of survivors had at least one unmet medical information need [14]. Lack of knowledge about their treatment late effects for adult childhood cancer survivors has also been linked to fear and anxiety of cancer recurrence [15]. Moreover, unless they have been to a dedicated survivorship clinic and have received this detailed information, they likely do not have easy access to this information when they may need it. Childhood cancer survivors may be at even greater risk due to their limited access to information, their age at original cancer diagnosis (too young to understand or to remember treatment), and the influence of parental guarding and parental decision-making [16]. These are all a cycle of compounding factors that lead to further barriers in pediatric cancer survivor education and can be a significant obstacle to seeking appropriate follow-up care [16]. Ways to improve survivor knowledge of their treatment and also increase compliance with long-term follow-up recommendations for lab work, clinical testing, and medical evaluations are a constant challenge for survivorship clinics [17].

**Fig. 1:**
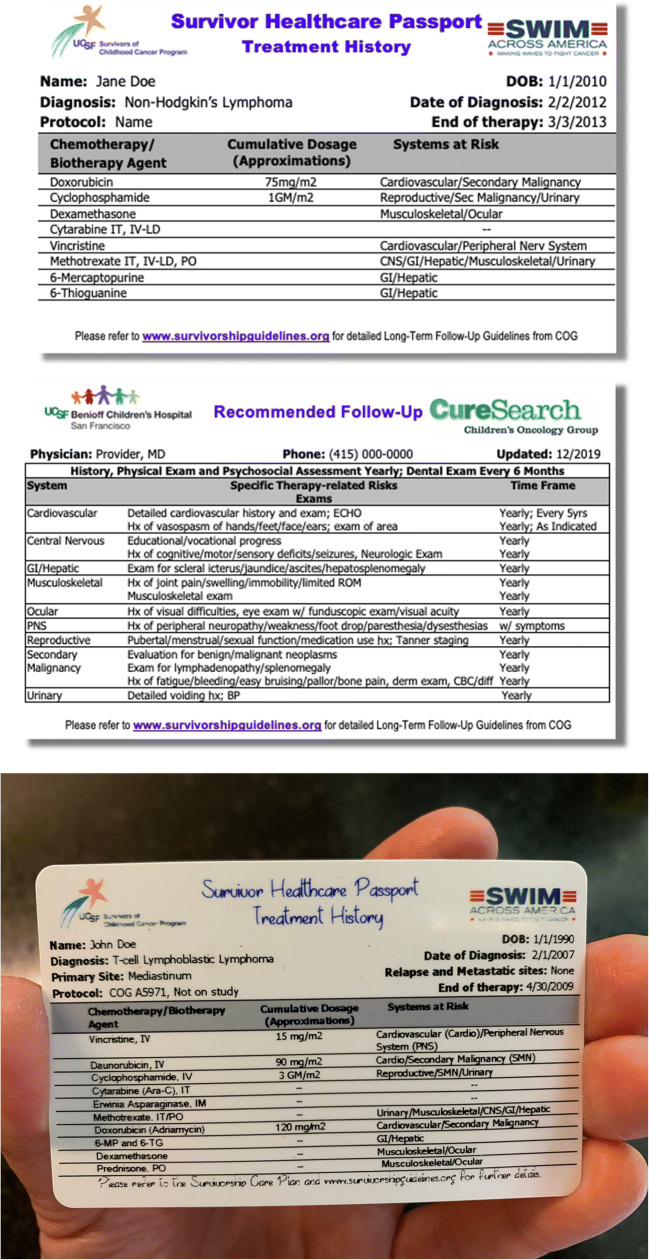
Portable, credit card cancer-related summary and follow-up care plan

The survivorship care plan (SCP) is an important and useful tool for cancer survivors, their families, and primary care physicians when patients transition to primary care from oncology [18]. The transition from primary oncology care to a primary care physician (PCP) can often be challenging for the cancer survivor as well as the PCP. A significant number of PCPs have reported a lack of knowledge in cancer late effects and caring for these patients [19]. The SCP contains relevant information for patients and other providers involved in their care, including a summary of their treatment (chemotherapy, radiation, and surgeries), dosing, and their associated late effects along with follow-up guidelines [20]. In efforts to help our clinic attendees retain information regarding their cancer diagnosis, treatment, and potential long-term side effects, along with recommendations for the monitoring of these systemic health risks (i.e., specialty referrals, ECHO, pulmonary function tests), the Survivors Program at the UCSF Benioff Children’s Hospital San Francisco developed the “Survivor Healthcare Passport.” This passport is based on the SCP that each patient also receives and is a durable, portable, wallet-sized plastic card that provides a summary of the patient’s diagnosis and treatment history along with a succinct assessment of late effects risks associated with their treatment (see Fig. 1). The passport includes recommendations for follow-up care based on the Children’s Oncology Group (COG) Long-Term Follow-Up Guidelines of Childhood, Adolescent and Young Adult Cancers along with our institutional practice guidelines [21]. Its value lies in its portability, ease of access, and succinct review of treatment and follow-up information.

To assess the benefits of this passport in educating patients and their families about their cancer diagnosis, therapy, and the long-term follow-up surveillance recommendations, we conducted a study where patients were randomized to either receive or not receive a passport and evaluated the impact of this passport on their specific cancer-related knowledge. The hypothesis was that the passport, as an educational tool, would improve patients’ knowledge of their treatment history and long-term follow-up needs.

## Methods

The UCSF Committee for Human Research approved this study for patients attending the Survivor Clinic. All patients regardless of age attending the Survivor Clinic for the first time and who had completed therapy at least 2 years prior to visit were eligible to participate. Those unable to speak or read English were excluded. The study was described to the patient and caregiver. Informed consent was obtained, and patients were randomized to one of two arms (arm A [passport given immediately after the first visit] or arm B [no passport given until after the completion of the 4-month last questionnaire]). Prior to their Survivor Clinic visit, all patients completed a baseline questionnaire (Q0) to assess knowledge of their cancer diagnosis, treatment history, and recommended follow-up (see Appendix 1). All patients also received verbal education and written information including COG Health Links, which are educational materials designed to enhance patient knowledge about specific systemic risks. All subjects then completed a questionnaire identical to the baseline questionnaire (Q0) at a short-term interval of 1 month (Q1) and again at a longer interval of 4 months (Q2) after their clinic visit. Both groups of patients received a copy of their treatment summary letter between completion of Q1 and Q2. Patients enrolled in arm B of the study received their passport after returning the Q2 questionnaire, ~ 4 months after their original visit. In the event that a completed Q1 or Q2 questionnaire was not received by the study center within 2 weeks of mailing, patients were contacted via telephone or email to ensure the questionnaire was received. A second copy of the questionnaire was provided to the patient if the document was lost. Questionnaires were mailed to patients via the US Post Office.

### Questionnaires and knowledge score

Each questionnaire received a score based on a scoring system consisting of a 100-point total for correct answers. Questions fell into one of the following four scoring categories: (1) diagnosis, (2) treatment, (3) risks, and (4) recommended follow-up. Up to 10 points were awarded for diagnosis-related answers, 40 points for treatment-related answers, and 50 points for various health- and follow-up-related answers.

The knowledge of diagnosis, treatment, risks, and follow-up care was measured by the sum of all points for each questionnaire that were completed by all participants. The knowledge score was assessed at baseline, 1-month, and 4-month intervals. The usefulness efficacy of the passport was evaluated by the change in the knowledge scores at 1 month from the baseline. The changes in the knowledge scores were imputed at two time points, month 1 and month 4, as percentage differences considering the score at the baseline to be 100%.

A responder was defined as a patient whose percent change in the knowledge score increased 50% or greater at 1 month or 4 months. The persistence effect of using the passport was evaluated by the comparison of the percent change in knowledge scores between month 1 and month 4.

## Methods and variables

The primary objective of the study was to determine whether the use of the passport improved the participant’s knowledge of diagnosis, treatment, risks, and follow-up care. The primary endpoint was the proportion of participants showing more than 50% improvement on the knowledge score from baseline to month 1 (short interval) or month 4 (long interval). A secondary endpoint was knowledge gain defined as greater than 20% improvement in their knowledge score.

The sample size was calculated based on Fisher’s exact test of difference between study groups on change of knowledge scores at 1 month or at 4 months. An effect size of 30% was assumed to be clinically significant. Using 90% power and assuming 10% attrition, it was calculated that at least 45 evaluable subjects were needed per group.

All patients who were randomized were included in the primary analysis population. As of intent-to-treat principle, no imputation was made to any missing score at the study time point; the number of patients in select subgroups varies in the analysis. Those who only completed the baseline questionnaire were not included in subsequent analyses. Descriptive statistics were used to summarize all endpoints as defined. Demographic, baseline characteristics, and patient disposition parameters (such as number randomized and number completing the study) were also summarized by descriptive statistics (Stata, version 15.0). In addition to the comparison of proportions, we have compared the average baseline score along with the change in the knowledge scores in month 1 and in month 4.

## Results

A total of 126 patients were enrolled, but only 110 completed at least two of the three questionnaires. This analysis includes 46 patients (42%) who were enrolled on arm A and received the passport and includes 64 patients (58%) who were enrolled on arm B and did not receive the passport. Clinical characteristics of the enrolled patients included in this analysis can be viewed in Table 1. Over half of the patients who participated were male (65%; *n* = 72) and white (66%; *n* = 73). There were no significant clinical differences between patients enrolled on arm A (passport) and those enrolled on arm B (no passport).

**Table 1:** Characteristics of the subject population

	Arm A (passport) *n* = 46		Arm B (no passport) *n* = 64	
Gender				
Female	19	41%	20	31%
Male	27	59%	44	69%
Diagnosis				
Leukemia	20	43%	20	31%
Lymphoma	6	13%	8	13%
CNS tumor	4	9%	6	9%
Neuroblastoma	3	7%	6	9%
Retinoblastoma	2	4%	0	–
Renal tumor	1	2%	3	5%
Bone tumor	2	4%	12	19%
Soft tissue sarcoma	5	11%	5	8%
Germ cell tumor	1	2%	3	5%
Other tumor	1	2%	0	–
Non-malignant diagnosis	1	2%	1	2%
Cancer therapy received				
Chemotherapy	46	100%	63	98%
Radiation therapy	25	54%	30	47%
Surgery	17	37%	35	55%
	Arm A		Arm B	
Mean age at study	22.6		23.3	
Mean age at diagnosis	8.1		9.7	
Mean time since diagnosis	14.5		13.6	

**Fig. 2:**
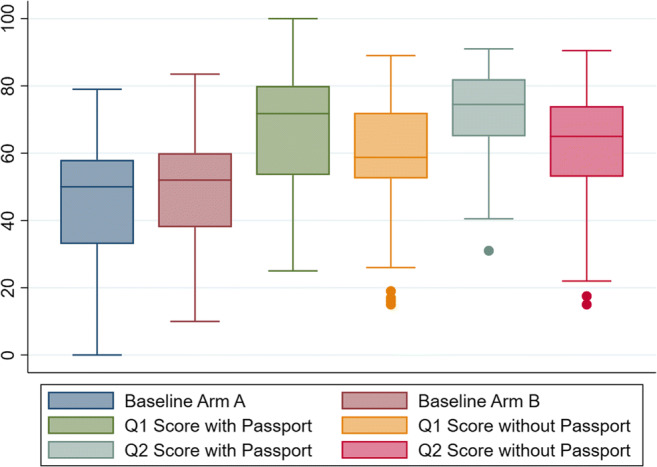
Questionnaire score distribution (box plot) at baseline, 1-month, and 4-month intervals

The mean score for the baseline questionnaire score in arm A was 45.4 (95% confidence interval, 39.7–51.2). The mean baseline questionnaire score for arm B was 49.0 (95% confidence interval, 44.0–53.9). The distribution of scores for the baseline and follow-up questionnaires is shown in Fig. 2. Between the baseline questionnaire and 1 month after the clinic visit (Q1), 45.2% of patients who received a passport during their survivor clinic visit (arm A) demonstrated a greater than 50% improvement in score compared with only 24.6% of patients without a passport (arm B) (Table 2, *p* = 0.034). The majority of those with a passport (55.8%) showed a greater than 20-point knowledge gain in their knowledge score compared with 26.2% of those without a passport between the baseline questionnaire to the questionnaire 1 month after the clinic visit.

**Table 2:** Knowledge score change

Score change	Passport Arm A (*n* = 46)	No passport Arm B (*n* = 64)	*p* value
Baseline to short interval questionnaire			
< 20 points	19 (44.2%)	45 (73.8%)	0.002
≥ 20 points	24 (55.8%)	16 (26.2%)	
< 50% improvement	23 (54.8%)	46 (75.4%)	0.034
≥ 50% improvement	19 (45.2%)	15 (24.6%)	
Baseline to long interval questionnaire			
< 20 points	14 (35.9%)	40 (74.1%)	0.001
≥ 20 points	25 (64.1%)	14 (25.9%)	
< 50% improvement	21 (55.3%)	41 (75.9%)	0.044
≥ 50% improvement	17 (44.7%)	13 (24.1%)	
Overall (between any questionnaire)			
< 50% improvement	14 (43.8%)	33 (68.75%)	0.003
≥ 50% improvement	18 (56.2%)	15 (31.25%)	

Similar benefits were seen with extended follow-up. After 4 months, 44.7% of patients with a passport had a knowledge score improvement of over 50% compared with only 24.1% of patients without a passport (*p* = 0.044). Ultimately, over half of those with a passport (56.2%) showed greater than 50% improvement in score between any of the two questionnaires, while less than half of patients without a passport (31.25%) demonstrated a 50% improvement between any questionnaires (*p* = 0.003). Overall, an overwhelming majority (84.8%) of patients with a passport showed improvement in their knowledge score of more than 20 points between any of the questionnaires, compared with 52.1% of patients without a passport (*p* value = 0.016).

## Discussion

The purpose of the Survivor Clinic at the UCSF Benioff Children’s Hospital is to educate patients and families about late effects and empower them to manage their healthcare after cancer treatment. It is the responsibility of healthcare providers to ensure patients and families have the best survivorship care planning resources available to do this. The future healthcare needs of survivors including tests, scans, and organ evaluations are often coordinated with their PCP. An easy follow-up care plan for survivors is needed, not only for PCPs but also for other potential specialists who may be involved in their care such as dentists, reproductive health specialists, cardiologists, and ophthalmologists. After the survivorship clinic visit, a detailed 8–12-page survivorship care plan is sent to the primary care physician and also sent to the patient for their records. It is detailed, yet lengthy. At the end of the visit, the patient is also given the Survivorship Healthcare Passport that is a detailed, concise credit card–sized card that can be kept in their wallet. It is easily accessible and has a full summary of their treatment, potential late effects, and recommended testing and frequency. The recommendations are patient-specific and are based on their treatment, dosing, age at diagnosis, and other concomitant therapies (i.e., radiation, surgery). It can be accessed in a matter of seconds to present to an ER triage nurse, the ophthalmologist evaluating for cataracts, or a physician assessing for scoliosis.

This study demonstrates that this passport can effectively enhance a survivor’s knowledge of their cancer diagnosis, treatment, and follow-up needs. As might be expected, knowledge improved in both groups of patients that either received a passport or did not initially receive one. Therefore, attending our survivor clinic helps improve a survivor’s knowledge and highlights the benefit of a dedicated survivor program. The added benefit of the portable passport card, with over 30% increase in average knowledge score, may improve compliance with long-term follow-up.

A written SCP should be individual to each survivor and the conversation should be ongoing regarding the physical, psychosocial, and neurocognitive effects [22]. This credit card–sized passport summarizes this care plan and is a useful tool for practitioners in survivorship clinics to educate patients and their families about their diagnosis, treatments, and follow-up needs. Reviewing this information with patients/families should reinforce the importance of having a full written treatment record along with the utility of the portable passport. Discussions should occur regarding how the passport can facilitate this management in different healthcare scenarios. The challenge for survivor clinics is translating this acquired knowledge into action for the benefit of patients and families. Many survivors may not seek medical follow-up and the likelihood of long-term follow-up may decline over time from completion of therapy [23].

While we did not test the question of technology vs. tangibility, this research begs the question as to whether a technologically based format would be more or less effective, or whether providing both formats to patients would be optimal. In addition to this passport’s ease of accessibility, a tangible plastic card may be just as, or even more, beneficial as a phone app or web-based version. Numerous studies show that when you read a text on paper, then your understanding is deeper and longer lasting than if you read that same text on a computer. For example, a study in Norway concluded that students who read texts in print scored significantly better on the reading comprehension test than students who read the texts digitally [24]. The importance of a mental map of the text in its entirety makes it easier for the brain when a person can feel and see the document; this mental map is more important if the text is complex and quicker navigation through the text is facilitated when you are able to understand relationships and context [24].

There is utility to a web-based option for storage and retrieval of survivorship late effects as there are web-based care plans available including “Passport for Care” that studies have shown improved communication about potential late effects and closer adherence to guidelines [25]. Another study conducted in the Netherlands which looked at the efficacy of a web-based SCP, a kind of virtual passport, found positive results regarding improvement in patient knowledge [26]. However, as of now, these are restricted to the web and not yet available in a mobile app version. Despite the hundreds of mobile phone apps for cancer survivors that have been developed over the past several years, few have been successful due to lack of patient involvement in the development and testing stages [27]. Using this knowledge, we are continuing to move forward with plans to develop a survivorship mobile phone app that will automatically update the patients’ personal late effect recommendations and potentially send reminders to patients about appointments and tests that are due. However, the credit card–sized passport will continue to be distributed to our patients and families as a tangible product. A study examining the strengths and weaknesses of each approach, web-based vs. mobile phone app vs. tangible card, would be of interest.

We have identified a number of limitations in this study. The first being that the patient population was relatively small and homogeneous. The Survivor Clinic at UCSF sees a wide range of patients, many of whom speak no or very limited English. This population was excluded from the study and may be a source of bias. However, it is likely that this population would have the same need for such an educational tool in their own language. Further investigation regarding language barriers and cancer-related knowledge is needed. The period of the evaluation of the passport’s effect was limited to a short 4-month interval; the longer term effect is not known. The study was also limited by a built-in bias of the increased incentive for patients who did not receive the passport to complete the questionnaires. Patients who were randomized to not receive the passport had more incentive to complete all questionnaires than those who received their passport, as only after they completed all three questionnaires would they receive their passport. This likely explains the higher number of patients (64 in arm B [no passport] versus 46 in arm A [passport]). Also, the font on the passport is small and may be difficult to read for those with limited vision unless a copy machine to magnify the text is available.

## Conclusion

Survivors deserve a concise summary of their therapy and of their long-term follow-up needs. The portable, wallet-sized passport is one method to enhance their cancer-related knowledge. Tools like the passport will aid in helping survivors and survivorship care teams improve long-term follow-up care. Having a tangible card in their wallet is a format for quick and easy access for survivors to view late effects and follow-up needs.
